# A Targeted Literature Review and Patient Focus Groups to Develop Recommendations for Reporting Patient-Reported Measures to Patients and Consumers in Clinical Quality Registries

**DOI:** 10.1177/11786329261477135

**Published:** 2026-09-07

**Authors:** Rasa Ruseckaite, Chethana Mudunna, Ilana Ackerman, Belinda Gabbe, Susannah Ahern

**Affiliations:** 1 School of Public Health and Preventive Medicine, 2541 Monash University, Melbourne, VIC, Australia

**Keywords:** clinical registries, patient reported measures, reports, improvement, patients, consumers

## Abstract

**Background:**

Clinal Quality Registries (CQRs) often collect patient reported measures (PRMs) for the purpose of reporting these data to clinicians. By incorporating PRM data, CQRs and participating healthcare providers can gain a more comprehensive understanding of patient experiences and outcomes to inform both individual care and broader health service improvements. However, many CQRs do not routinely provide patient access to PRM data or report these data back to patients.

**Objectives:**

To understand how PRMs captured in CQRs should be reported to patients, and to develop a guide for reporting registry-collected PRMs to this population.

**Methods:**

First, a targeted literature review was undertaken, involving a structured search of the scientific literature to identify evidence on patient preferences regarding the reporting of PRMs. Data were extracted and managed using Microsoft Excel. Second, focus group discussions were conducted with 15 registry consumers to explore their PRM information needs, and preferences for how registry-collected PRM data should be reported.

**Results:**

The literature review identified 23 studies and found that many patients preferred to receive their own PRM data. Access to these data helped them better understand their health, support discussions with clinicians, and feel more empowered in their care. Graphical displays and lay summaries were most preferred. Focus group participants also valued receiving aggregate reports and being informed about how their data were used and recommended clear terminology and accessible formats for diverse audiences.

**Conclusion:**

Based on the findings from this study, we developed a guide with practical resources, examples, and guidance for CQRs on how to engage with patients in the PRMs’ reporting process. The goal of this document is to support transparency in PRMs reporting, patient engagement with CQRs, and the real-world use of this data for improving healthcare quality.

## Introduction

Patient reported measures (PRMs) are captured in clinical settings to examine the impact of treatment and care on a patient’s health-related quality of life (HRQoL), daily functioning and symptom experience.^1,2^ These measures allow for the comparison of health outcomes across different healthcare providers or institutions, facilitating benchmarking and identifying and promoting best practices. PRMs can be applied at multiple levels, ranging from informing individual clinical encounters to monitoring performance at the service or system level.

In Australia, the National Clinical Quality Registry (CQR) Program (the Program) aims to improve the quality of health care and ensure better health outcomes for Australian patients. As part of this Program, the Australian Government Department of Health, Disability and Ageing is leading a range of activities under A National Strategy for CQR and Virtual Registries 2020-2030 (the Strategy).^3^ Increasing the use of PRMs in national CQRs is a key priority of the Strategy. Registries, which collect, aggregate and analyse patient data to improve quality of care for patients, often collect PRMs for the purpose of reporting these outcomes solely to clinicians and for quality improvement (QI) purposes.^4,5^

While the need for patient feedback is recognised as crucial for improving healthcare quality and patient satisfaction,^6^ the actual practice of providing feedback to patients is often limited.^7^ Previous studies have indicated that this may act as a barrier to CQRs achieving high PRMs response rates,^8^ where patients are not valued around their wellbeing, with a resulting decrease in PRMs response rates over time.^9,10^ Furthermore, the lack of feedback to patients undermines the overall PRMs reporting process where these data can be used to facilitate patient-centred care.^11^ It is therefore important that relevant feedback which acknowledges patient perspectives is provided, although there is little clarity on how this feedback can be provided, what feedback should be provided and how often. Much of the current research around PRM reporting has focused on improving how PRM data should be presented.^1,12-16^ There is limited understanding of how PRMs should be reported to patients when captured in CQRs.

To address these gaps, a co-design process, where patients and consumers are actively involved in informing PRMs data reporting activities is important.^17^ In an effort to standardize PRMs reporting processes for CQRs, we developed a PRMs reporting guide for clinicians and registry operators.^18^ This guide aims to streamline data reporting in CQRs for QI purposes. However, it may not be suitable for reporting PRMs to patients due to several reasons. First, the technical language and processes mentioned in the guide may be difficult for consumers to understand or engage with due to different levels of health literacy. Second, the guide does not include patient or consumer perspectives,^19^ which is crucial for continued consumer participation in CQRs.^18^

The aim of this study was to explore patient and consumer perspectives on the reporting of CQR-collected PRM data and to develop preliminary principles and recommendations to guide reporting practices within Australian CQRs.

## Methods

The research process was iterative. A targeted review of the literature was performed by systematically searching databases for available national and international scientific literature on PRM data reporting including presentation approaches.

Online focus groups were organised with patients and consumer representatives from Australian CQRs to explore their experiences and views with regard to PRMs data reporting in CQRs. Participant recruitment was guided by the study aim of obtaining a range of patient and consumer perspectives from Australian CQRs. Consistent with qualitative research methodology, the sample size was determined pragmatically based on available participants, representation across different registry types, and the feasibility of conducting multiple focus groups within the study timeframe.

The research team comprised researchers with expertise in CQRs, PRMs, health services research and qualitative methods. Data interpretation was undertaken collaboratively through regular team discussions to challenge assumptions and ensure that emerging themes reflected participant perspectives. The researchers were not involved in the clinical care of participants.

### Literature

#### Search Strategy

A structured literature search was undertaken using predefined search terms and eligibility criteria. The review process included database searching, duplicate removal, independent screening, full-text assessment and data extraction. Given the targeted nature of the review, the objective was to identify relevant evidence to inform the qualitative component of the study rather than to comprehensively map all available literature. To identify relevant studies, we searched MEDLINE and EMBASE databases. Additionally, a grey literature search was performed in Google Scholar (www.googlescholar.com). Studies with all types of designs were eligible if they discussed patient and consumer involvement in the reporting and/or interpretation of PRM data. No date limits were set for the search. RR and CM developed a search strategy using Medical Subject Heading (MeSH) keywords and free text search terms (Table 1). The terms were combined using Boolean operators. Studies were excluded if they were published in a language other than English or did not discuss patient involvement. For each publication, abstracts and full articles were obtained. Reference lists of the included studies were examined for other relevant studies.

**Table 1.** Search Strategy for PRMs Data Reporting to Patients

Domain 1 and domain 2	
Domain 1: Patient Reported Measures	Patient Reported Outcome Measures/**OR** (patient report* outcome* measure* adj4 regisr*).mp. OR ((patient reported outcomes or patient-reported outcomes or patient reported outcome measures or patient-reported outcome measures or PROMs) adj3 (register* or registr*)).mp **OR Patient Reported Experience Measures/**
	**OR**
	Patient Reported Measures
Domain 2: Consumer Involvement	consumer/**OR**
	patient/**OR**
	patient involvement* interpretation*.mp. **OR**
	consumer involvement* interpretation*.mp. **OR**
Domain 1 AND Domain 2	

#### Selection of Articles

The study selection process comprised four phases. First, two researchers (RR and CM) conducted the literature search, exporting records to EndNote and uploading them to Covidence (Version 2), where duplicates were removed. Second, titles and abstracts were independently screened for eligibility, with discrepancies resolved through discussion. Third, full texts were independently assessed against inclusion and exclusion criteria, and disagreements were resolved by the study team. Finally, a data extraction template was developed in Covidence, and relevant data were extracted.

#### Data Synthesis and Analyses

For each article, the following data of the full-text articles were extracted in Microsoft Excel by spreadsheet: Author; Year and country of the study; Aim of study; Study Design; Population description and setting; Materials and methods; and Study outcome.

The data synthesis was done by CM and RR and consisted of searching for consistency of patterns across the extracted data in Microsoft Excel and making comparisons between the extracted data of the studies with similar methodologies. Similarities were observed as a pattern and supplemented with any important distinguishing information.

Literature findings were grouped to two categories:1) Visualisation of PRMs reports, and 2) Outcomes and impact made by PRM data reporting. Data were then narratively synthesised to understand how PRMs data are analysed and presented to patients.

### Focus Groups

#### Participants

Patient participants were recruited through social media, CQR consumer reference groups, snowball sampling and through contacts of the research team.

Eligible participants were adults (aged ≥18 years) who were patients, carers, consumer representatives, or members of consumer advisory groups affiliated with an Australian CQR. Participants were required to be able to provide informed consent and participate in an online focus group conducted in English. Individuals who were unable to provide informed consent, were not affiliated with a CQR, or were unable to participate in the focus group discussions in English were excluded from the study.

Participants received a AUD20 gift voucher in recognition of their time and contribution to the study.

#### Data Collection

Five focus groups were conducted in February 2025 at Monash University via Zoom teleconference software. An exploratory qualitative project methodology was employed to understand information needs, personal perspectives regarding PRM data collection, interpretation and reporting preferences in clinical practice and CQRs.^20^

A focus group protocol was developed that included a topic guide detailing prompt questions for participants (Supplementary Material 1). Key topics for focus groups were based on the findings from the literature review. They included: patient experiences with PRMs, patient perspectives regarding PRM data collection and reporting in CQRs, and a discussion of already existing recommendations for PRMs data reporting. Additionally, samples of hospital-level CQR PRMs reports were reviewed to further discussion on PRMs presentation.

#### Data Analysis

All audio recordings were transcribed, and thematic analysis was conducted. Transcripts were read for familiarity, meaningful segments were coded, and codes were organized into themes, which were then cross-checked against the original data. Trustworthiness and validity were considered by assessing the credibility, transferability, dependability, and confirmability of the findings. The Consolidated Criteria for Reporting Qualitative research (COREQ) were used to guide the reporting of the qualitative findings^21^ (Supplementary Material 2).

### Principles and Recommendations

The themes from the focus groups were discussed internally among the research team and combined with the findings from the literature review to inform development of principles and recommendations for PRM data reporting.^22^ Findings from the literature review and focus groups were integrated and compared to identify areas of convergence and divergence. Combining evidence from these complementary sources provided a broader understanding of how patients interpret PRM data and how PRM information may be presented and explained to this audience.

The study was conducted between September 2024 and February 2026, including the literature review, focus group data collection, analysis, and development of recommendations.

## Results

### Literature Review: Study Selection and Characteristics

**Figure 1.**
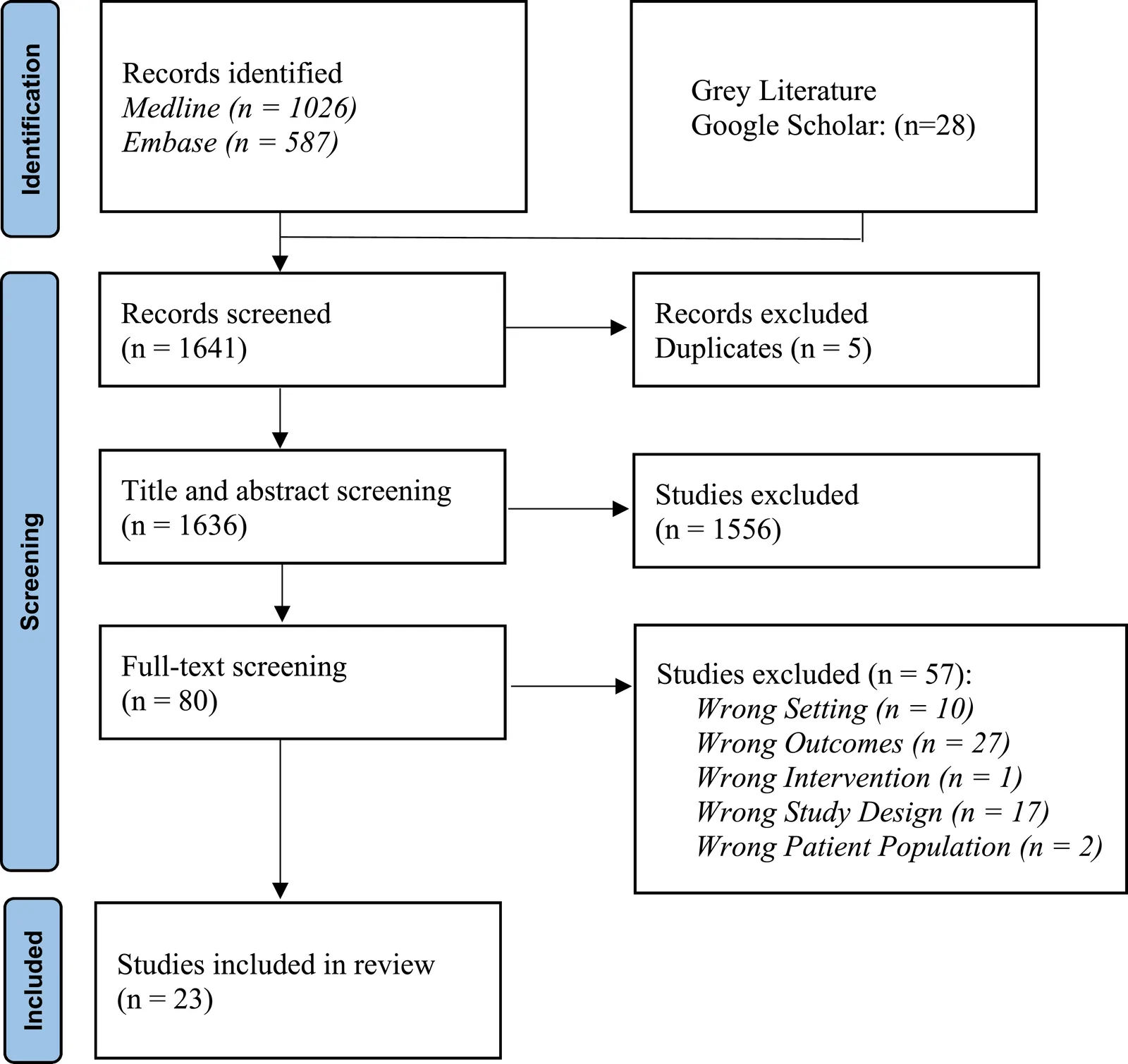
PRISMA flow diagram of the search process

The search of databases MEDLINE and EMBASE yielded 1613 documents (Figure 1). A further search through grey literature identified 32 documents. After removing duplicates, 1636 documents were screened for titles and abstracts. Of those, full copies of 76 relevant articles were retrieved. The screening of full-texts resulted in 23 relevant studies, of which data were extracted and analysed.

Studies included in the final review were published between 2003 and 2023. Of the 23 publications, 14 (80.9%) were published in the United States, and two (8.7%) in the United Kingdom. The remaining seven studies were published in the Netherlands, Canada, Italy, Australia and Israel or were multi-national. Ten studies (43.5%) were qualitative, eight (34.8%) used a cross-sectional or mixed methods study design, one (4.3%) was an implementation study and three (13.1%) were randomized control trials (Table 2).

**Table 2.** Targeted Literature Review of Patient Involvement in PRM Reporting and Visualisation Studies

Author, year, country	Aims of the study	Population description	Materials used	Outcomes
Brundage et al.^23^ 2003, Canada	To explore cancer patients’ attitudes and preferences regarding the communication of HRQoL information in clinical trials.	33 patients in 3 Canadian cancer centres, identified from upcoming appointment lists or patient registry	10 visual formats for communicating HRQoL information to initiate group discussions used for focus group discussions	Preferred formats for communicating HRQoL data included using visual aids or written explanations
Brundage et al.^24^ 2015, United States	To evaluate how different graphic formats of PROs are interpreted by patients	50 cancer patients recruited from 8 academic and community institutions	Semi-structured interviews and surveys regarding: 1) Format preferences and interpretation accuracy (line graphs, bar charts), 2) Objective vs preference: accuracy of interpretation vs subjective preference, 3) Comprehension of multidimensional data across multiple domains, 4) Ease of use of the graphical displays	Line graphs were preferred over bar charts and tables
Damman et al.^25^ 2018, Netherlands	To explore how PROMs are interpreted and utilized in medical consultations for shared decision-making	80 patients with Parkinson’s disease recruited through a local Parkinson’s cafe, an advertisement on the website of the Dutch Association of Parkinson’s Disease, and Neurology department	Surveys to assess different presentation formats: 1) Correct interpretation of PROMs information, 2) Perceived usefulness of the PROMs information, 3) Participants’ attitude towards using the PROMs information in routine medical consultations and 4) Preferred ways to receive PROMs information.	Patients were positive about using PROMs in routine medical consultations. They preferred aggregated PROMs for seeing treatment decisions. Line graphs presented the most accurate interpretation of PROMs
Franklin et al.^26^ 2021, United States	To evaluate the effectiveness of tailored PRO-based shared decision reports, the study developed a decision tool for patients with advanced knee and hip osteoarthritis	7891 orthopaedic patients	Web-based system in order to generate a tailored report at the point of clinical decision-making. Iterative patient interviews defined report content and visual display.	Patients successfully complete the pre-visit PRO assessments. The web-based system components allow real-time individualized reports to patients.
Galper et al.^27^ 2019, Israel	To explore the implementation of PROs to improve healthcare quality and enhance patient engagement through shared decision-making	PCa, acute coronary disease and cataract surgery patients recruited at 20 active clinical areas	The process involved selecting and reporting right tools and adapting them to clinic culture and patient needs, including language barriers.	The introduction of the patient portal was a significant development, leading to a higher engagement rate and better tracking of outcomes through reporting the data to patents
Gerteis et al.^28^ 2007, United States	To explore 1) how visual cues help consumers interpret the displays of nursing home measures, (2) whether reporting formats other than the bar charts enhance comprehension, and (3) to what extent consumers’ self-reported preferences for one format or another correlated with accurate interpretation	90 individuals, 45 - 75 years of age	The study tested alternative data displays, with limited explanatory text, 3. Categories of templates: 1) Evaluative templates using symbols or words; 2) Numeric templates; 3) Graphic templates. Two questions were asked: 1) a warmup question that required reading the information displayed and 2) a question that required some interpretation of the data displayed in the template.	Fewer than half of respondents accurately interpreted bar graphs. Respondents made fewest errors on templates using words
Grossman et al.^12^ 2018, United States	To design and test an interface for engaging heart failure patients in completing PRO surveys using data visualization	25 heart failure patients, using purposeful sampling on age and race, from a cardiac inpatient unit and an ambulatory cardiac clinic at an urban academic medical centre	Symptoms interface, Health IT Usability Evaluation Scale (Health-ITUES) and survey regarding storyboarding techniques to detail patients’ interactions with the interface and determine the necessary components. Interview topics included application’s general usefulness, usefulness for patient provider communication, helpful and unhelpful features, and suggested changes.	Patients found the interface helpful in understanding symptoms, though comprehension challenges were noted. Visualization of their results improved understanding and engagement.
Hawley et al.^13^ 2008, United States	To evaluate the ability of six graph formats to impart knowledge about treatment risks/benefit to low and high numeracy individuals	2412 participants from a panel of Internet users	Online hypothetical medical decision-making scenario: participants were told that there were two pills they could take to unclog their arteries and possibly avoid needing bypass surgery (called Pill A and Pill B). However, each pill had a risk of mild headaches and severe nausea. Participants were then randomized to receive risk/benefit information (1) bar graph; (2) pictograph; (3) modified pictograph (‘sparkplug’); (4) pie chart; or (5) modified pie graph (‘clock graph’); and (6) table.	Pictographs were the best format for communicating probabilistic information to patients, particularly among lower numeracy individuals. Providers can consider using pictographs to communicate risk and benefit information to patients of different numeracy levels.
Hildon et al.^29^ 2012, United Kingdom	To explore patients’ perceptions of various formats (e.g., tables, bar charts) and content (e.g., uncertainty, statistical significance) used to display quality indicators of healthcare providers	6 focus groups, with 45 participants. Recruited through Arthritis Care and the Royal College of Surgeons Patient Liaison Group.	Focus group guides used to facilitate discussion, focusing on how participants understood various visualizations and what they thought of different data formats for comparing healthcare providers. The participants were shown different data formats and were asked to provide feedback on their usefulness and ease of understanding.	Tables with star ratings were the most preferred format, as they were easier for patients to understand and process. Familiar formats like bar charts led to misinterpretations. Icons (like stars) and colour coding (such as traffic lights) helped improve comprehension.
Izard et al.^30^ 2014, United States	To address deficiencies in PCa cancer care that limit discussion of the HRQOL impacts of treatment	50 patients from local support groups and urology clinics. Eligible patients were >21 years old, had PCa irrespective of stage or time since treatment, and could read and understand English.	Prototype dashboards and questions that measured comprehension and perceived usefulness. Each dashboard illustrated sample HRQoL data for a fictitious patient scenario based on assessment of example patients with the Expanded Prostate Cancer Index.	Developed the design concept of a dynamic HRQoL dashboard that permits a base patient-centred report in bar chart format that can be toggled to other formats and include error bars that frame comparison group scores.
Jayakumar et al^31^ 2020, United States	To assess the effect of an AI-enabled patient decision aid that includes education, preference assessment, and personalized outcome estimations on decision quality, patient experience, functional outcomes, and process-level outcomes	Randomized clinical trial at a single US academic orthopaedic practice included 129 new adult patients presenting for osteoarthritis-related knee pain	The primary outcome was decision quality, measured using the Knee OA Decision Quality Instrument (K-DQI). Secondary outcomes were collaborative decision-making (assessed using survey), patient satisfaction with consultation (using a numerical rating scale), Knee Injury and Osteoarthritis Outcome Score Joint Replacement (KOOS JR) score, consultation time, TKR rate, and treatment concordance.	An AI-enabled decision aid significantly improved decision quality, satisfaction, and physical limitations without significantly impacting consultation times.
Kuijpers et al.^32^ 2016, Europe, international	To investigate patients’ and health professionals’ understanding of and preferences for different graphical presentation styles for individual level of QLQ-30	548 international patients with cancer	Patients completed the QLQ-C30 to become familiar with the instrument. Based on their randomization, patients received one of 5 frequently used graphical presentation styles: non-coloured and coloured bar charts and line charts, and a heat map.	Patients’ objective and self-rated understanding were similar for 5 graphical presentation styles, although they had a slight preference for bar graphs. Self-rated understanding was substantially higher and did not differ significantly between professions or graphical presentation styles.
Liu et al.^33^ 2019, United States	To develop a dashboard that could facilitate conversations about PROs and that would be acceptable to a wide range of patients, including English and Spanish speakers and patients with adequate or limited health literacy	Rheumatoid arthritis: 6 focus groups involving 25 patients	Patient focus groups to assess prototype mock ups. They were formed based on patient language preference (English and Spanish) and health literacy level.	Many patients found the dashboard difficult to understand at first glance. This initial confusion was more common among limited health literacy patients but not exclusive to them. Several patients did not notice or understand the longitudinal nature of the data. At least one limited health literacy patient found the design not pleasing. Some patients did not understand the dashboard at all until focus group leaders or other patients explained the sections.
Lombi et al.^34^ 2023, Italy	To examine patients’ experiences with e-PROMS, particularly their perspectives about its usefulness and its implications for the clinical encounter with their doctors	19 cancer patients	Semi-structured interviews: questions on the feasibility and acceptability of the routine use of e-PROMs in clinical cancer settings, difficulties participants encountered, information and help received about e-PROMs, and participants’ perceptions of the impact of the e-PROM on the clinical consultation	Some participants saw significant benefits to the use of e-PROMs in routine clinical practice in cancer settings: helped patients feel that they were supported, particularly regarding emotional and psychological aspects of their illness; e-PROMs were found to enhance patient’s awareness about their own symptoms.
Loth et al.^35^ 2016, Austria	To investigate cancer patients' understanding of graphical presentations of longitudinal QLQ-C30 scores	40 brain tumour patients	Descriptive bar charts: patients were asked whether the displayed questionnaire results matched their personal perception of symptoms and functional health during the period displayed.	Patients are able to understand their QOL results when presented graphically and interpret absolute scores and changes over time. Patients consider the QOL trajectories to adequately match their own perceptions of their health status. If using questionnaires such as the QLQ-C30 with different scale directions, provision of detailed information on the meaning of high and low values is essential.
McNair et al.^36^ 2010, United Kingdom	To assess patients’ understanding of multidimensional PROs in a graphical format	132 Esophageal and gastric cancer patients.	Survey regarding graphical representation of the data: treatment changes in a single PRO over time; different PRO with the direction of treatment reversed; graph showing divergent, then convergent PROs in one dimension; graph with increased complexity showing PRO changes over 18 months with the two treatments having a different impact on physical function; graph with treatment	High level understanding of simple graphs. Fewer patients could understand complex graphs.
Smith et al.^14^ 2016, United States	To improve formats for presenting individual-level PRO data (for patient monitoring) and group-level PRO data (for reporting comparative clinical studies)	39 cancer eligible patients >21 years, able to communicate in English	Iterative stakeholder driven process with workgroups- semi-structured one-on-one interviews. PRO presentation assessed: conveying score meaning and directional inconsistency; concerning scores for individual-level PROs and identifying important between-group differences for group-level PROs	Variations in interpretation accuracy demonstrate the importance of presenting PRO data in ways that promote understanding and use. The iterative stakeholder-driven process, developed improved PRO data presentation formats is needed
Snyder et al.^16^ 2017, United States	To test approaches for presenting PRO data to improve interpretability	Cancer: 10 interviews, 627 surveys.	Surveys with prepared formats for data presentation regarding the direction of the scores.	Some formats were helpful in identifying whether higher scores were good or bad. For example, the green shading led to easier understanding. The threshold lines were considered helpful, especially with the arrow reinforcing the direction. Higher 5 better directionality was both more accurately interpreted and more likely to be rated clear.
Snyder et al.^15^ 2019, United States	To develop stakeholder engaged, evidence-based recommendations for PRO data display for the three above applications to promote understanding and use	Cancer: 28 - Delphi round 1, and 26 - Delphi round 2	Consensus panel. Two issues were addressed for three applications: directionality of PRO score display and conveying score meaning	Consensus panel recommended showing line graphs of scores over time and including some indication of possibly concerning results in absolute terms (where evidence exists to support the concerning PRO score range). Consensus panel agreed that PRO data display should accommodate both normed and non-normed scoring and that display of the norm is optional, depending on the trade-offs between added interpretive value vs. potentially greater complexity.
Snyder et al.^17^ 2022, United States	To conduct user testing to assess comprehension, utility, and preference of longitudinal PRO visualizations designed for prostate cancer survivors with limited literacy	Prostate cancer participants aged 61-77 (M=69), of whom half were African American.	Meter (A), Words (B), Comic (C), and Emoji (D) prototype to assess comprehension, utility, and preferences among 4 prototype PRO visualizations. User testing involved individual sessions lasting 60-90 mins to assess the prototypes and literacy for each participant.	Meter had the best gist comprehension and was preferred. Emoji was also preferred, had the highest verbatim comprehension, and highest rated utility, including helpfulness, confidence, and satisfaction. Meter and Words both rated mid-range for utility, and Words scored lower than Emoji and Meter for comprehension. Comic had the poorest comprehension, lowest utility, and was least preferred
Stern et al,^37^ 2022, United States	To explore patients’ perspectives on the benefits of receiving feedback on PROMs in the context of a web-based personalized decision report to guide care for their hip or knee osteoarthritis.	25 patients with hip or knee osteoarthritis	Qualitative descriptive interview study was nested in a pragmatic clinical trial of a personalized report, which includes descriptive PROM scores and predicted postoperative PROM scores. Patients completed a semi-structured interview within 6 weeks of an office visit with an orthopaedic surgeon	Patients identified actual and hypothetical benefits of receiving feedback on PROM scores in the context of a web-based decision report, including advantages for those who had already made a treatment decision before seeing the surgeon.
Stern et al,^38^ 2023, United States	To describe examples of opportunities, challenges, and priorities throughout PROM implementation to equitably advance value-based care at both the patient and population level	Review of studies in patients with musculoskeletal diseases	Review off opportunities, challenges, barriers and enablers.	Balancing standardization with tailored strategies can enable the large-scale implementation of PROMs while optimizing care processes and outcomes for all patients.
Tolbert et al.^39^ 2018, United States	Toi identify the association of PRO score directionality, score norming, and other factors on a) how accurately PRO scores are interpreted and b) on how clearly, they are rated to be by patients, clinicians, and PRO researchers	Cancer: 629 surveys, 10 cognitive interviews	Questionnaire: three accuracy questions about the line graph type to which they were randomized. After completing the accuracy questions, participants rated the line graph formats clarity. The last question provided an opportunity for open-ended feedback about the graphs in a text box	Overall, line graphs received the most negative feedback and the majority of comments focused on the issue of directionality

e-PROM: Electronic Patient Reported Outcome Measure, Health-ITUES: Health- IT Usability Evaluation Scale, HRQoL: Health Related Quality of Life, PCa: Prostate Cancer, PRO: Patient Reported Outcome, PROM: Patient Reported Outcome Measure, QLQ-30: Quality of Life Questionnaire-30, QLQ-C30: Quality of Life Questionnaire- Core 30.

### Visualisation of PRMs Reports

Fourteen studies (60.9%) identified graphical PRMs presentation as the most preferred option.^13,15,16,24-26,28,30-32,35-37,39^ These included bar charts, pictographs and line graphs.^35^ In contrast, participants in two studies misinterpreted PRMs when presented graphically, primarily due to limited health literacy.^29,33^ Participants from four studies (17.4%) preferred visual or written aids to assist with PRMs interpretation.^12,17,23,38^ In one study, patients preferred to receive PRMs scores summarised in tables as they were “easier to understand”.^29^ In another study, a meter format was employed with minimal use of text, colour, and arrows to describe PRMs data.^17^ A “meter presentation” of refers to visualising health metrics on a gauge or dial-like chart, similar to a speedometer or energy meter, to show a current value against a target or scale.^40^ Dashboard reporting provides a visual, centralized display of key health data and often accessible in real-time received mixed responses. Participants with limited health literacy found this method to be challenging and less acceptable.^33^

### Outcomes and Impact Made of PRM Data Reporting

Several positive outcomes from reporting PRMs to patients were identified. First, receiving PRMs reports made patients feel supported.^26,34,37,38^ Second, by identifying ways patients could interpret PRM data, clinicians were able to communicate better risk-benefit information.^13^ Third, patients were better able to reflect on their health and identify problems which they would like to discuss with clinicians during consultations.^23,41^ Consequently, clinicians and service providers were able to improve quality of care for patients by tailoring care to their needs.^25,^^34^ Finally, by identifying preferred methods for PRM data presentation and interpretability, patients were able to match their perceptions of their health status and better understand their health conditions.^4,12,35^ For example, when patients were able to interpret their health changes over time, identifying the direction of trajectory assisted to match their own beliefs to the data presented.

### Focus Group Discussions

Fifteen people participated in five focus groups. The mean age of the participants was approximately 48 years, and 13 (87%) of the participants were female. The participants represented five Australian CQRs collecting medical device, cancer and chronic disease outcomes. In line with the topic guide, the main topics discussed at the focus groups included: 1) PRMs collection and reporting, 2) Privacy and consent for PRM data reporting, 3) PRMs data visualisation and presentation for patients, and 4) Accessibility of PRM data reports.

#### PRMs Collection and Reporting

Focus group participants identified three approaches to support patient understanding of PRM data: (1) clinician contact regarding PRMs, (2) direct reporting of PRM results to patients, and (3) real-time PRM data reporting. For clinician contact, participants suggested a registry-level triage system to flag safety or well-being concerns, prompting clinician follow-up (“*something [could be] flagged as a concern…that the registry would pick it up initially*” FGD3P1). Others preferred direct outreach from data collectors, e.g., “…*you’ve reported a very poor quality of life in this aspect…How can we help you…?*” (FGD4P3).

For direct reporting, participants emphasized a feedback loop to inform patients how their PRM data were used (“*there should be a loop that comes back to [patients]…*” FGD3P1). They also supported dashboard-based PRM reports, noting these should be “*more realistic*” (FGD4P2) and highlighting that digital, centralized systems are more efficient than paper formats, particularly in technologically advanced settings (*“…there must be a technical person that can put that data…into an application*” FGD4P23).

#### Privacy and Consent for PRM Data Reporting

Participants emphasized the importance of privacy and informed consent when completing PRM questionnaires, including whether data would be identified or de-identified and how it would be protected. Many preferred contact from their clinician (“*would feel more comfortable if the contact came from [the clinician]*” FGD3P1), while some expressed concern that data might be used solely for research rather than shared with their doctor (“*…you assume it’s going for research purposes…not back to your doctor*” FGD3P2).

#### PRM Data Visualisation and Presentation

Some participants suggested presenting PRM scores like blood test results, using reference ranges and showing previous results to highlight values outside the normal range (“*flag it in red if it’s outside…*,” FGD3P1). They also recommended visual formats such as pictograms, e.g., coloured stick figures representing percentages of patients experiencing outcomes (“*I like to see 50 little figures… 25 are coloured differently*,” FGD3P2).

Participants noted that the brightest or most colourful elements draw attention (“*the most colourful…is what [they] are going to assume is important*,” FGD3P3). Colours should be chosen carefully: red to indicate problems (e.g., high pain scores) and blues or greens for positive outcomes. Suggestions included shading contrasts or simple red–green coding (“*something that’s a problem…could be red, and if it’s good [then] green*,” FGD3P2), while acknowledging that some colours may signal danger to patients (FGD3P1).

Participants emphasized that PRM results should be “*really simple*,” “*easy to digest*,” and in “*layman’s terms*” (FGD3P1, FGD3P3). Line charts were preferred for showing trends over time, while bar charts were useful for comparing specific values. They also suggested audio or verbal formats to explain results without technical jargon (“*if they can listen…rather than read all of that technical jargon*” (FGD3P1)).

#### Accessibility of PRM Data Reports

When appropriate, CQRs should tailor individual PRMs reports for people from diverse backgrounds, including culturally and linguistically diverse and Indigenous populations. This is to ensure that data are accessible, inclusive and culturally appropriate to these individuals. Participants suggested considering *“someone who may be non-English speaking, different cultural background”* or *“Aboriginal and Torres Strait Islander people and how do they want to get this information”* (FGD3P3). Study participants also considered vision impaired individuals and their accessibility to PRMs, for example, through the use of a *“screen reader”* (FGD3P3). However, also it was also highlighted that for visually impaired individuals it would be *“very hard to read certain text on colours. Some colours are easier than others”* (FGD4P2) and *“red can be highly triggering”* (FGD4P3).

Focus group participants expressed the need to consider literacy levels. People can be *“computer literate”* (FGD2P2) and *“might have some sort of a learning disability”* (FGD2P3). In addition, the study participants identified language accessibility as an important consideration. For example, *“terms that are accepted within medical settings, for instance, dysfunction would need to possibly be a more acceptable term to a patient who might be reading that and find things like that a little bit triggering”* (FGD3P1), or that some individuals see themselves *“as patients, not consumers. It’s a term that I don’t think we should be using in this space*” (FGD3P1).

### Principles and Recommendations for Reporting PRMs Captured by CQRs

Draft principles and recommendations for reporting PRMs to consumers and patients were developed with input from patients and consumer representatives, informed by findings from the literature review and focus group discussions. These recommendations are intended as preliminary guidance to support PRM reporting practices within CQRs. The following sections were included (Table 3): 1) Patient involvement in CQR PRMs programs, 2) PRMs selection, 3) PRMs administration, 4) Ethics and consent, 5) General PRMs data reporting principles, 6) PRMs in CQR annual reports, 7) PRMs in site/provider reports, 8) PRMs data visualisation for patients/registry participants, 9) Individual patient data reporting, 10) PRMs reports for benchmarking and quality improvement, 11) Real-time PRMs data reporting and dashboards.

**Table 3.** Summary of the Recommendations for Reporting PRMs to Patients

Section title	Description
Patient involvement in CQR PRMs programs	Recommendations on involving consumers throughout PRMs planning, governance and report development, including representation of culturally diverse and Indigenous communities.
PRMs selection	Guidance on selecting PRMs instruments and item banks, with patient involvement in instrument selection.
PRMs administration	Recommendations on the timing, frequency, and response rates of PRMs collection.
Ethics and consent	Guidance on consent, privacy, data security, and ethical considerations in PRMs reporting.
General PRMs data reporting principles	Recommendations on presenting PRMs results in plain language, using clear terminology, multiple communication formats, and reports that are easy for patients to understand and use.
PRMs in CQR annual reports	Recommendations for including aggregate PRMs data in annual reports, standalone reports, or infographics.
PRMs in site/provider reports	Guidance on incorporating PRMs data into site and provider reports, aligned with clinical reporting cycles.
PRMs data visualisation for patients/registry participants	Recommendations on presenting PRMs data using clear visualisations, benchmarks, and clinically meaningful interpretations.
Individual patient data reporting	Recommendations on providing patients with personalised PRMs results, including graphical presentation, explanations of score meaning, change over time, and guidance on discussing results with clinicians.
PRMs reports for benchmarking and quality improvement	Recommendations on using PRMs data to monitor outcomes over time, benchmark performance, and support quality improvement.
Real-time PRMs data reporting and dashboards	Recommendations on providing secure, timely access to personalised PRMs results through digital dashboards where feasible, while considering privacy, technical capability, and clinical workflows.

A draft of the recommendations was provided to participants and the Australian Government Department of Health, Disability and Ageing for their review, with feedback incorporated into the final version of the document. The recommendations are publicly available on the department’s website.^42^

## Discussion

This project has developed a set of guiding principles and recommendations for CQR reporting of PRMs data to patients and consumers. This document serves as a supplementary resource to our guide ‘Using Patient Reported Measures in Clinical Quality Registries for Healthcare Improvement: A Guide’, which provides guidance for the use and reporting of PRMs within CQRs to drive healthcare improvement.^43^

Findings from the literature review and focus groups highlighted patients’ preferences for PRM presentation, reporting, accessibility, and privacy, emphasizing the need for transparent communication to support consumer engagement in CQR PRM programs. The findings can be interpreted through the lenses of patient-centred care, health literacy, and shared decision-making. Participants emphasised the need for accessible language, meaningful feedback, transparency regarding data use, and support to understand and act on PRM information. Due consideration to effective data presentation in PRM reports was highlighted as of high importance. Participants emphasized the need to cater to a diverse range of consumers who may not have similar levels of literacy or understanding of PRMs data and clinical terminology. Plain language summaries, with culturally sensitive terminology, and visual aids such as pictograms or colours were most preferred.

Participants emphasized that meaningful feedback loops and iterative reporting can empower patients by providing insights into their care, supporting shared decision-making, and improving outcomes. However, participants also highlighted the importance of ensuring that reports are simple, accessible and appropriately explained, recognising that poorly presented information may be confusing, burdensome or distressing for some individuals. Such loops also demonstrate the value of PRM completion, potentially increasing patient engagement in CQR PRM programs. These findings underscore international research that advocate for the involvement of patients in PRMs reporting.^12,27,41^ However, prior research has focused on clinical trials and reporting PRMs to professional audiences, while this study related to reporting PRMs for consumers from clinical registries.

The findings also show consumer interest in benchmarking outcomes and experiences when presented accessibly, highlighting the need to enhance dashboards, individual reports, and other PRM reporting methods tailored to patient needs. While participants generally viewed access to PRM data positively, unintended consequences may arise if reporting is not carefully implemented. The literature review found that some patients, particularly those with lower health literacy, may have difficulty interpreting PRM reports. Complex displays, unclear scores, and information about poor health status may cause confusion, anxiety, or distress, especially when clinical support or contextual explanations are lacking.

Participants highlighted the importance of language, cultural appropriateness, and health literacy in PRM reporting. However, CALD populations and Aboriginal and Torres Strait Islander peoples were not represented in the focus groups, limiting the applicability of findings to these groups. Given the known barriers to PRM participation among culturally diverse and marginalised populations, an equity-focused approach to reporting is needed. This should include culturally adapted, accessible, and plain-language reporting, as well as co-design with underrepresented communities to ensure PRM data are relevant, meaningful, and accessible to all patients.^44^

Drawing on the literature, consumer perspectives, and expert input, these recommendations provide a practical framework to support PRM reporting in CQRs. However, their relevance, priority, and feasibility will vary according to registry objectives, patient populations, available resources, and organisational context.

The recommendations are not intended as a prescriptive checklist. While some strategies, such as using plain language, ensuring transparency, providing accessible data visualisations, and involving consumers in reporting activities, may be achievable for most registries, others, including real-time dashboards, individualised reporting, and clinical escalation pathways, may require substantial infrastructure and investment. A staged implementation approach may therefore be appropriate, beginning with foundational practices that support accessibility and patient engagement.^45^ In addition, it is crucial to acknowledge that not all the recommendations listed in the guide would be applicable to everyone. For example, reporting data to individual patients can be costly due to high administrative overheads, significant personnel costs and person-hours required for data preparation, analysis and reporting. The recommendations for registries to develop and manage clinical escalation pathways may likely not be feasible due to a lack of resources, the need for local context and clinical judgment, and challenges in data collection and implementation. Developing escalation pathways involves significant logistical challenges and potential risks, such as misinterpretation of patient data and ensuring timely, appropriate responses.^46^

### Strengths and Limitations

Several measures were undertaken to enhance the rigour of this study. Credibility was supported through systematic data collection and thematic analysis, while dependability was strengthened using a clear study protocol and consistent interview procedures across focus groups. Confirmability was achieved by ensuring correct focus groups transcriptions, that all participant responses were included in the thematic analysis and regular research team meetings to validate the process and analysis.

Several limitations should be acknowledged. The literature review was not specific to PRM reporting in registries and may have missed relevant studies. The focus groups included a small, predominantly female sample of highly engaged consumers, which may limit the generalisability of findings to the broader registry population. Formal saturation was not assessed. In addition, participants from CALD backgrounds and Aboriginal and Torres Strait Islander communities were not represented, limiting the applicability of findings to these populations. Finally, the recommendations should be viewed as preliminary guidance rather than definitive standards. Further validation and implementation research are needed to assess their feasibility, acceptability, and impact across different registry settings.

## Conclusion

In conclusion, this study generated a set of guiding principles and recommendations for reporting PRM data from CQRs to consumers, to be made available via the Australian Government Department of Health, Disability and Ageing’s National CQR Program website.^42^ These recommendations are expected to evolve with advances in PRM reporting, practical feedback from implementation, and further research on their use and impact. Future research should evaluate the feasibility, acceptability and effectiveness of these recommendations across diverse registry settings and patient populations, including culturally and linguistically diverse communities and Aboriginal and Torres Strait Islander peoples. Further work is also needed to examine the impact of different PRM reporting approaches on patient understanding, engagement, health literacy and healthcare decision-making.

## Supplemental Material

Supplemental Material - A Targeted Literature Review and Patient Focus Groups to Develop Recommendations for Reporting Patient-Reported Measures to Patients and Consumers in Clinical Quality Registries Supplemental Material for A Targeted Literature Review and Patient Focus Groups to Develop Recommendations for Reporting Patient-Reported Measures to Patients and Consumers in Clinical Quality Registries by Rasa Ruseckaite, Chethana Mudunna, Ilana Ackerman, Belinda Gabbe and Susannah Ahern in Health Services Insights.

Supplemental Material - A Targeted Literature Review and Patient Focus Groups to Develop Recommendations for Reporting Patient-Reported Measures to Patients and Consumers in Clinical Quality Registries Supplemental Material for A Targeted Literature Review and Patient Focus Groups to Develop Recommendations for Reporting Patient-Reported Measures to Patients and Consumers in Clinical Quality Registries by Rasa Ruseckaite, Chethana Mudunna, Ilana Ackerman, Belinda Gabbe and Susannah Ahern in Health Services Insights.

## Data Availability

Due to confidentiality and data use agreements, the focus group datasets analysed in the current study are not publicly available. Requests to access the datasets for bona fide research purposes should be directed to the corresponding author.*
