# Supplementary material for: A Targeted Literature Review and Patient Focus Groups to Develop Recommendations for Reporting Patient-Reported Measures to Patients and Consumers in Clinical Quality Registries
Source: Health Serv Insights. 2026 Sep 7;19:11786329261477135. doi: 10.1177/11786329261477135 (PMC13554694; doi:10.1177/11786329261477135)
Supplement: Supplementary file 1 [file sj-pdf-1-his-10.1177_11786329261477135.pdf]

Supplementary Material 1: Reporting Patient Reported Measures from Clinical Quality Registries. Guide for consumers

Key topics for focus group discussions

- Consumer experience with PRMs
    - *How often have you discussed/seen the PRM-results, and in what setting?*
    - *How did you experience the discussion about the PRM-results? What went well when discussing the PRM-results, and what could be improved?*
    - *Do you contribute PRMs to your registry? How is the data captured and reported?*
    - *What are your expectations for the PRM-results, and what did it bring you?*
    - *What should be the purpose of discussing/reporting the PRM-results in the registry?*
    - *How can PROM-results contribute to the treatment or wellbeing of the patient?*
    - *What should be done after collecting PRMs in the registry?*
- Consumer perspectives regarding PRMs data collection and reporting in CQRs
    - *Enabling physicians to monitor health status more effectively?*
    - *Improving self-awareness?*
    - *Improving treatment and care?*
    - *Contributing to clinical research?*
    - *Contributing to benchmarking and quality improvement?*
- Discussion of already existing recommendations for clinicians
    - *Usefulness of the existing guide?*
    - *Are they comprehensible and whether they could be adapted to consumers?*
    - *What key changes would be required?*
- Developing PRMs reporting guide for consumers
    - *Content for reporting: longitudinal comparisons, stratification of the data?*
    - *Outliers?*
    - *Presentation of the aggregated data?*
    - *Tables/summaries/charts/graphs*
    - *Scores and their presentation?*
    - *Real time data and dashboards*
    - *Narrative and wording*
- Addressing cultural sensitivities in the reports
